# Multi-Modality Imaging for Prevention of Coronary Artery Disease and Myocardial Infarction in the General Population: Ready for Prime Time?

**DOI:** 10.3390/jcm11112965

**Published:** 2022-05-24

**Authors:** Daan Ties, Paulien van Dorp, Gabija Pundziute, Erik Lipsic, Carlijn M. van der Aalst, Matthijs Oudkerk, Harry J. de Koning, Rozemarijn Vliegenthart, Pim van der Harst

**Affiliations:** 1 University Medical Center Groningen, Thorax Centre, Faculty of Medicine, University of Groningen, 9713 GZ Groningen, The Netherlands; d.ties@umcg.nl (D.T.); p.van.dorp@umcg.nl (P.v.D.); g.pundziute@umcg.nl (G.P.); e.lipsic@umcg.nl (E.L.); 2 Erasmus Medical Center, Department of Public Health, Erasmus University, 3015 CE Rotterdam, The Netherlands; c.vanderaalst@erasmusmc.nl (C.M.v.d.A.); h.dekoning@erasmusmc.nl (H.J.d.K.); 3 Institute for Diagnostic Accuracy, University of Groningen, 9713 GZ Groningen, The Netherlands; oudkerk@i-dna.org; 4 University Medical Center Groningen, Department of Radiology, Faculty of Medicine, University of Groningen, 9713 GZ Groningen, The Netherlands; r.vliegenthart@umcg.nl; 5 University Medical Center Utrecht, Department of Cardiology, University of Utrecht, 3584 CX Utrecht, The Netherlands

**Keywords:** coronary artery disease, population, screening, early diagnosis, myocardial ischemia, coronary atherosclerosis, myocardial perfusion imaging, computed tomography, risk factors, atherosclerotic cardiovascular disease

## Abstract

Cardiovascular disease (CVD) remains a leading cause of death and disability worldwide. Acute myocardial infarction (AMI) causes irreversible myocardial damage, heart failure, life-threatening arrythmias and sudden cardiac death (SCD), and is a main driver of CVD mortality and morbidity. To control the forecasted increase in CVD burden for both the individual and society, improved strategies for the prevention of AMI and SCD are required. Current prevention of AMI and SCD is directed towards risk-modifying interventions, guided by risk assessment using clinical risk prediction scores (CRPSs) and the coronary artery calcium score (CACS). Early detection of more advanced coronary artery disease (CAD), beyond risk assessment by CRPSs or CACS, is a promising strategy to allow personalized treatment for the improved prevention of AMI and SCD in the general population. We review evidence for further testing, beyond CRPSs and CACS, and therapies focusing on promising targets, including subclinical obstructive CAD, high-risk plaques, and silent myocardial ischemia. We also evaluate the potential of multi-modality imaging to enhance the conduction of adequately powered trials to provide high-quality evidence on the impact of add-on tests and therapies in the prevention of AMI and SCD in asymptomatic individuals. To conclude, we discuss the occurrence of AMI and SCD in individuals currently estimated to be at “low-risk” by the current strategy based on CRPSs, and methods to improve prevention of AMI and SCD in this “low-risk” population.

## 1. Introduction

Acute myocardial infarction (AMI) is one of the main drivers of cardiovascular morbidity and mortality worldwide. AMI often occurs in asymptomatic individuals with pre-existent subclinical coronary artery disease (CAD) and has severe consequences for these previously healthy individuals by causing irreversible myocardial damage, heart failure, life-threatening arrhythmias and sudden cardiac death (SCD). Clinical risk prediction scores, such as the Pooled Cohort Equations (PCE) and the Systematic Coronary Risk Estimation 2 (SCORE2), were proved to adequately predict AMI and SCD at population level [1,2]. Professional practice guidelines recommend initiating risk modifying treatment for the prevention of AMI and SCD in asymptomatic individuals, guided by cardiovascular risk assessment using these clinical risk prediction scores [3,4]. Over the last few decades, several non-traditional risk markers were proposed to improve risk assessment by clinical risk prediction scores, including carotid intima-media thickness, ankle–brachial index and the coronary artery calcium score (CACS). Of these risk markers, CACS was shown to provide the best discrimination of risk [5]. Clinical practice guidelines currently recommend performing the CACS in borderline- or intermediate risk cases in the United States (i.e., >7.5–≤20% PCE risk) or in cases with a SCORE2 risk around treatment decision thresholds in Europe, to reclassify risk up- or downward and to guide treatment by lifestyle interventions alone or in combination with preventive drug therapy [3,4]. Strategies to prevent AMI and SCD were successful in reducing cardiovascular mortality and morbidity in the past [6], but despite implementation of these strategies, cardiovascular disease (CVD) still remains a leading cause of death and disability worldwide [7,8]. In addition, CVD-related healthcare costs are forecasted to steeply increase in Western societies [9]. Further development of strategies to more effectively prevent AMI and SCD is warranted to fight the increasing CVD burden for both the individual and society.

## 2. Targets beyond Clinical Risk Factors and Coronary Calcium Burden

Rather than focusing on clinical risk factor burden or total plaque burden by CACS, targeting disease directly causing AMI and SCD could improve the prevention of AMI and SCD. There is conflicting evidence on which atherosclerotic plaques exactly cause AMI and SCD. An older meta-analysis from 1995 suggested that the majority of AMIs is caused by so-called “vulnerable” or non-obstructive plaques (i.e., with luminal narrowing <50%) [10]. However, data for this meta-analysis were gathered from only a limited number of studies with small sample sizes. Importantly, these studies measured the degree of luminal narrowing on index coronary angiograms that were acquired months to years prior to AMI or SCD, and were therefore unable to determine a causal relationship between the degree of luminal narrowing and AMI or SCD. Accelerated plaque progression and high-risk features of plaques, such as positive remodeling, high necrotic core volume and thin fibrous cap are currently well-recognized characteristics of atherosclerotic plaques that cause AMI and SCD [11,12]. Severe luminal narrowing is another strong predictor of plaque rupture causing AMI and SCD. Recent angiographic studies measuring degree of luminal narrowing in infarct-related arteries after thrombo-suction, fibrinolysis or spontaneous reflow revealed that severe coronary stenosis is present just prior to the plaque rupture [13,14,15]. Importantly, in a post-mortem study, ruptured plaques and high-risk plaques with intact fibrous caps showed similar histological features, but ruptured plaques were on average larger in size [16].

## 3. Coronary Computed Tomography Angiography for Identification of the At-Risk Population

In contrast to clinical risk prediction scores and CACS, coronary computed tomography angiography (CCTA) can provide information on plaque location and characteristics, including luminal narrowing and high-risk plaque features. In a population-based study from Sweden, Bergström et al. performed CCTA in 25,182 individuals without known CAD and discovered obstructive CAD (diameter stenosis ≥ 50%) in 5.2% of the population [17]. In an American population-based study performing CCTA in 2,491 individuals free of CVD, a 6% prevalence of significant coronary artery stenosis (diameter stenosis ≥ 50%) was observed [18]. In the same study, high-risk plaque features were discovered in 7% of study participants [18]. Whether the early detection of obstructive CAD improves risk stratification beyond CACS remains debatable. A sub-study in 3217 asymptomatic individuals from the CONFIRM registry showed that the presence of obstructive CAD (diameter stenosis ≥ 50%) on CCTA provided incremental prognostic utility for the prediction of mortality and non-fatal myocardial infarction for individuals with CACS 101–400, but not for those with lower or higher CACS [19]. In another study, among 23,759 symptomatic patients the presence of obstructive CAD was not predictive of AMI, stroke and mortality beyond plaque burden in most CACS categories [20]. However, the predictive ability of obstructive CAD specifically for AMI and SCD was not reported in this study [20]. Several drug therapies were shown to potentially enhance plaque stabilization and healing [12]. Early identification of high-risk plaque beyond CACS could allow more personalized treatment in these individuals for more effective prevention of AMI and SCD. The FACTOR64 study evaluated screening by CCTA and subsequent aggressive risk factor control or invasive coronary angiography versus standard care with aggressive risk factor control in patients with diabetes mellitus, and discovered that CCTA screening did not lower the CVD event rate [21]. However, large randomized controlled clinical trials (RCTs) comparing CCTA-guided preventive measures against an untreated group in asymptomatic individuals free of diabetes mellitus have not been performed. Definite proof on the benefit or harm of early CCTA-guided prevention, with accurate identification of plaques prone to rupturing and precisely targeted therapy for these plaques, are currently lacking. Importantly, CCTA is associated with a relatively high radiation burden (2.5–5 mSv) [22]. Radiation-induced cancer is a risk of using CCTA in preventive strategies in the general population and should be closely monitored by future RCTs evaluating CCTA-guided therapy in a preventive setting.

## 4. Myocardial Perfusion Imaging for Identification of the At-Risk Population

### 4.1. Myocardial Ischemia

Obstructive CAD (i.e., atherosclerotic plaque with a high degree of luminal narrowing) can limit coronary perfusion and can cause ischemia of underlying myocardial tissue. However, a mismatch between stenosis severity estimated by CCTA and presence of myocardial ischemia exists, partly caused by partial volume effects causing inaccurate estimation of stenosis severity [23]. Myocardial perfusion imaging (MPI) by single-photon emission computed tomography (SPECT), positron emission tomography (PET) or cardiac magnetic resonance (CMR) can accurately assess the presence of myocardial ischemia and could provide incremental prognostic value beyond coronary artery calcium (CAC) scoring or CCTA. Recently, a large retrospective study showed that silent myocardial ischemia strongly predicts AMI and SCD in 1027 asymptomatic individuals [24]. Myocardial ischemia is a well-known target for therapy in symptomatic CAD. Several years ago, some RCTs and a meta-analysis of these RCTs have shown that the cardiovascular event rate is reduced by ischemia-guided revascularization in symptomatic patients with CAD [25,26,27,28]. Since then, research evaluating the treatment of myocardial ischemia was mainly centered around the method of choice to treat symptomatic myocardial ischemia. Ischemic size of ≥10% of the left ventricle (i.e., moderate–severe myocardial ischemia) was suggested as the turning point at which revascularization improves clinical outcome, compared to optimal medical therapy (OMT) alone [29]. In contrast to these trials, the COURAGE trial failed to show a reduction in cardiovascular events by revascularization, as compared to OMT in patients with evidence of moderate–severe myocardial ischemia [30]. More recently, the ISCHEMIA trial randomizing 5179 patients with moderate–severe myocardial ischemia to either revascularization or OMT did not show a significant reduction in the composite of death, myocardial infarction and stroke by revascularization, compared to OMT [31]. Irrespective of the presence of symptoms, professional practice guidelines currently recommend treating large areas of myocardial ischemia (>10% of left ventricle) by OMT alone or combined with revascularization to improve prognosis [32,33]. However, data on treatment of asymptomatic or silent myocardial ischemia are scarce. In the COURAGE Nuclear sub-study, a reduction in ischemic size of ≥5% was beneficial for the outcome [34], but whether reducing ischemic size translates into improved clinical outcomes for asymptomatic individuals, as compared to no treatment, either by OMT alone or in combination with revascularization, remains to be proven. Bansal et al. performed screening for silent myocardial ischemia by SPECT-MPI in 1123 asymptomatic diabetics in the DIAD trial and discovered no difference in clinical outcome for screening versus no screening, but this trial was underpowered for studying this endpoint [35]. No other adequately powered RCTs evaluating whether MPI screening for ischemia in asymptomatic individuals improves outcome compared to no screening were performed to date. The EARLY-SYNERGY trial, evaluating outcomes after screening for silent myocardial ischemia by CMR-MPI versus no screening in asymptomatic individuals with increased CACS will provide more clarity on this issue [36].

### 4.2. Unrecognized Myocardial Infarction and Reduced Left Ventricular Ejection Fraction

Besides the presence of ischemia, MPI can evaluate the presence of unrecognized myocardial infarction by late gadolinium enhancement imaging. In the SPINS study, performing CMR-MPI in 2349 patients, suspected of CAD, unrecognized myocardial infarction was present in approximately 15% of the patients and unrecognized myocardial infarction was strongly associated with recurrent AMI [37]. The ICELAND-MI study performing CMR-MPI in 935 patients suspected of CAD found a similar prevalence of unrecognized MI of 17% among patients, and a strong association of unrecognized myocardial infarction with mortality during the 10-years follow-up [38]. Secondary prevention by lifestyle modification and drug therapy were proved to reduce recurrent myocardial infarction and death rates and is recommended by professional practice guidelines [39,40]. Importantly, patients with unrecognized myocardial infarction exhibited lower use of preventive drug therapy, compared to individuals with recognized myocardial infarction in the SPINS study [37]. The impact of secondary preventive therapy on outcomes in those with early detected previously unrecognized myocardial infarction in the general population remains to be determined by large secondary prevention trials. By determining left ventricular ejection fraction (LVEF), CMR-MPI also allows for early detection and treatment of unrecognized heart failure and the indication for implantable cardioverter-defibrillators to prevent lethal arrythmias in those at risk [41,42], but the benefits of routine screening for LVEF by MPI in asymptomatic individuals also remain to be further studied. 

## 5. Multi-Modality Imaging for Identification of the At-Risk Population and Increased Statistical Power of Trials

**Figure 1:**
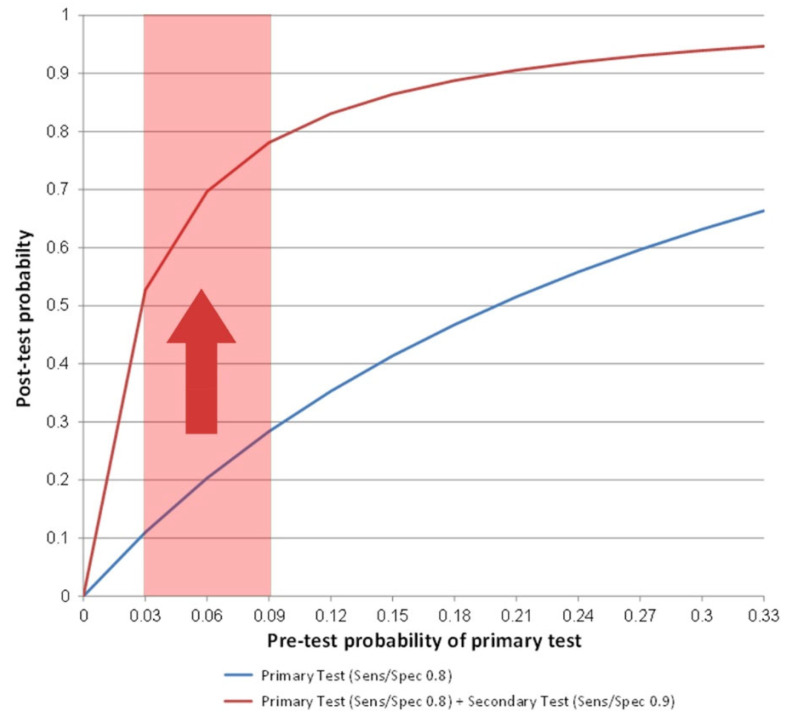
Theoretical framework of multi-modality testing to early diagnose advanced CVD. Pre-test probability (i.e., prevalence of disease) and post-test probability of disease presence given a positive screening test, calculated by Bayes Theorem, for an approach with one test only (sensitivity and specificity of 0.8) (**blue line**) and for an approach combining a primary (sensitivity and specificity of 0.8) and a secondary test (sensitivity and specificity of 0.9) (**red line**). In the range of pre-test probabilities reflecting a screening setting with low-prevalent disease (light-red area), post-test probability is markedly increased after a positive secondary test in a multi-modality imaging approach compared to post-test probability after a single positive test result in a single-test approach (**red arrow**). Sens = sensitivity; Spec = specificity.

Many RCTs evaluating the efficacy of add-on tests and therapies for low-prevalent disease in asymptomatic individuals from the general population lack sufficient statistical power to reliably estimate the effect of these interventions. If only a single test is applied to a population with low pre-test probability of disease presence, post-test probability of disease presence is low, regardless of diagnostic test performance (Figure 1). A combination of two tests, in which the first test naturally increases the pre-test probability of the second test, substantially increases the post-test probability of disease presence after a positive secondary test (Figure 1). Cancer screening programs were already proven successful by combining primary testing to assess risk, and targeted secondary testing, to diagnose severe disease [43,44]. A CACS of 400 or higher as the study entry criteria was shown to maximally improve the efficiency and feasibility of RCTs evaluating the incremental efficacy of novel add-on strategies [45]. Interestingly, an increased CACS predicts the presence of obstructive CAD and high-risk plaque features on CCTA [17,18]. Increased CACS also predicted an increased ischemia prevalence on MPI in symptomatic patients [46]. Implementation of a multi-modality imaging strategy, with CACS as a gatekeeper to add-on tests and therapies, could enhance the conduct of adequately-powered RCTs for the appropriate evaluation of existing and new add-on strategies for improved AMI and SCD preventive practice. Performing diagnostic tests in a multi-modality imaging strategy will be associated with costs. Whether more powerful prevention of AMI and SCD, thus potentially minimizing the need for expensive AMI treatments and potentially reducing productivity losses, can be achieved by this strategy, and whether this results in a reduction of overall costs as compared to the current strategy with CRPSs remains to be investigated. To date, no large trials performing a head-to-head comparison of cost-effectiveness between multi-modality imaging and CRPSs alone were performed. The EARLY-SYNERGY trial will provide the first evidence on this topic [36].

## 6. Screening the General Population for Risk Status

AMI and SCD frequently occur in individuals who are classified as “low-risk” by clinical risk prediction scores, or who have remained unrecognized [47]. Several studies showed that individuals who are classified as “low-risk” by commonly used clinical risk prediction scores have coronary atherosclerosis, putting them at increased risk of suffering from AMI or SCD [48,49,50,51]. In line with these findings, clinical risk prediction scores were shown to exhibit moderate discrimination and calibration for AMI risk [52]. The prevalence of high CACS in individuals with “low-risk” ascertained by the new SCORE2 remains unknown. CACS-based initiation of risk-modifying interventions, for instance by statins, was proven effective for the prevention of AMI at population level, even in low-risk individuals [53,54,55]. Individuals from lower socio-economic groups are known to be underrepresented in CAD preventive care and remain unrecognized until suddenly facing AMI or SCD [56]. To date, it remains unclear if screening the general population by CACS, CRPSs or other tests results in improved clinical outcomes, and, if proven beneficial, whether it would be cost-effective. Screening the general population for cardiovascular disease is currently investigated by a population-based screening trial conducted in the Netherlands. The ROBINSCA (Risk Or Benefit IN Screening for CArdiovascular disease) trial randomly invited around 43,000 inhabitants from three regions in The Netherlands for participation in this trial and randomly allocated these individuals, if meeting the inclusion or exclusion criteria, to either CACS screening, CRPS (i.e., SCORE) screening or no screening [57]. Participants and their general practitioners were informed on the cardiovascular disease risk as estimated by CACS or CRPS and provided with recommendations on initiating preventive measures guided by this risk estimation. The primary objective of the ROBINSCA trial is to evaluate whether screening the general population for cardiovascular disease by either CACS or CRPS improves clinical outcomes compared to no screening. The results of the ROBINSCA trial should be awaited to draw some first conclusions on the potential benefit and cost-effectiveness of screening the general population for cardiovascular disease. Close evaluation of radiation-induced cancer incidence caused by CACS screening is essential, although improved computed tomography techniques have resulted in very low radiation doses associated with CACS and the radiation burden is now comparable to, for instance, screening mammography (<1 mSv) [58,59]. In a cross-sectional analysis, a first CACS scan at an age of approximately 42 years in men and 57 years in women resulted in a testing yield of 25% for detecting CACS > 0 [60]. If proven beneficial, further prospective research evaluating the optimal selection of CACS screening candidates to maximize the benefit of screening and to minimize potential harm are warranted.

## 7. Conclusions and Future Perspectives

Prevention of AMI and SCD by risk-modifying interventions (i.e., lifestyle modification and drug therapy) was proven to effectively reduce CVD morbidity and mortality in the past. However, the CVD burden for both the individual and society is forecasted to increase in the future. Improvement of current strategies for the prevention of AMI and SCD is required to fight the increasing CVD burden. Additional CAC scoring beyond standard risk assessment by clinical risk predictions scores is currently recommended in the case of intermediate or borderline CAD risk, to guide treatment strategy. To date, RCTs evaluating the impact of further screening for obstructive CAD or silent myocardial ischemia on clinical outcome have not shown a beneficial effect. However, these RCTs were mostly underpowered to study the impact of these screening approaches. Knowledge on which plaques are prone to rupturing causing AMI and SCD is still expanding and potential new targets for therapy might arise. RCTs applying a multi-modality imaging strategy, using CACS as a gatekeeper to further screening, are promising to evaluate the effect of further screening for obstructive CAD, silent myocardial ischemia and other new and existing targets for personalized preventive therapy with sufficient statistical power. Such RCTs should be conducted to provide definitive proof on the added value of add-on strategies beyond CAC scoring. Another potential target for improved AMI and SCD prevention lies in the current misclassification of many individuals as “low-risk”, thereby not receiving preventive treatment, and the many individuals remaining unrecognized and unassessed by current strategies. Screening the general population for CACS is a promising method to perform accurate risk assessment, apply appropriate preventive therapies and allow more widespread dissemination of prevention, supported by multi-modality imaging, among many more asymptomatic individuals. The ROBINSCA trial will provide more evidence on the benefit and harm of CACS screening versus SCORE screening and no screening. If proven beneficial, further research evaluating the optimal selection of CACS screening beneficiaries is essential to improve the chances of successful implementation of CAD screening in the future.

## Data Availability

Not applicable.
